# Characterizing Protein Interactions Employing a Genome-Wide siRNA Cellular Phenotyping Screen

**DOI:** 10.1371/journal.pcbi.1003814

**Published:** 2014-09-25

**Authors:** Apichat Suratanee, Martin H. Schaefer, Matthew J. Betts, Zita Soons, Heiko Mannsperger, Nathalie Harder, Marcus Oswald, Markus Gipp, Ellen Ramminger, Guillermo Marcus, Reinhard Männer, Karl Rohr, Erich Wanker, Robert B. Russell, Miguel A. Andrade-Navarro, Roland Eils, Rainer König

**Affiliations:** 1 Department of Mathematics, Faculty of Applied Science, King Mongkut's University of Technology North Bangkok, Bangsue, Bangkok, Thailand; 2 EMBL/CRG Systems Biology Research Unit, Center for Genomic Regulation, Barcelona, Spain; 3 Robert B. Russell, Cell Networks Protein Evolution, BioQuant, University of Heidelberg, Heidelberg, Germany; 4 Network Modeling, Leibniz Institute for Natural Product Research and Infection Biology - Hans Knöll Institute Jena, Jena, Germany; 5 Department of Knowledge Engineering, Maastricht University, Maastricht, The Netherlands; 6 Theoretical Bioinformatics, German Cancer Research Center, Heidelberg, Germany; 7 Department of Bioinformatics and Functional Genomics, Institute of Pharmacy and Molecular Biotechnology, BioQuant, University of Heidelberg, Heidelberg, Germany; 8 Integrated Research and Treatment Center, Center for Sepsis Control and Care (CSCC), Jena University Hospital, Jena, Germany; 9 Department of Computer Science V, Institute of Computer Engineering, University of Mannheim, Mannheim, Germany; 10 AG Neuroproteomics, Max Delbrueck Center for Molecular Medicine, Berlin, Germany; 11 Computational Biology and Data Mining Group, Max Delbrueck Center for Molecular Medicine, Berlin, Germany; Bar Ilan University, Israel

## Abstract

Characterizing the activating and inhibiting effect of protein-protein interactions (PPI) is fundamental to gain insight into the complex signaling system of a human cell. A plethora of methods has been suggested to infer PPI from data on a large scale, but none of them is able to characterize the effect of this interaction. Here, we present a novel computational development that employs mitotic phenotypes of a genome-wide RNAi knockdown screen and enables identifying the activating and inhibiting effects of PPIs. Exemplarily, we applied our technique to a knockdown screen of HeLa cells cultivated at standard conditions. Using a machine learning approach, we obtained high accuracy (82% AUC of the receiver operating characteristics) by cross-validation using 6,870 known activating and inhibiting PPIs as gold standard. We predicted *de novo* unknown activating and inhibiting effects for 1,954 PPIs in HeLa cells covering the ten major signaling pathways of the Kyoto Encyclopedia of Genes and Genomes, and made these predictions publicly available in a database. We finally demonstrate that the predicted effects can be used to cluster knockdown genes of similar biological processes in coherent subgroups. The characterization of the activating or inhibiting effect of individual PPIs opens up new perspectives for the interpretation of large datasets of PPIs and thus considerably increases the value of PPIs as an integrated resource for studying the detailed function of signaling pathways of the cellular system of interest.

## Author Summary

Mathematical models which aim to describe cellular signaling start from constructing an interaction network of effectors, mediators and their effected target proteins. Several developments came up making it easier to put these links together. Besides tediously assembling knowledge from textbooks and research articles, experimental high-throughput methods were established like Yeast-2-Hybrid assays or Fluorescence Emission Resonance Transfer. However, these methods do not elucidate the *effect* of such interactions. We aimed inferring if an interaction in a specific cellular context is rather activating or inhibiting. We used cellular phenotypes of a genome-wide RNAi knockdown screen of live cells to identify such activating and inhibiting effects of protein interactions. The rationale behind it is that activating protein interactions should lead to similar phenotypes when their respective genes are knocked down, whereas an inhibiting protein interaction should lead to dissimilar phenotypes. Exemplarily, we applied our method to a phenotype screen of perturbed HeLa cells. Our predictions effectively reproduced textbook relationships between proteins or domains when comparing the predicted effects with pairs of effectors, receptors, kinases, phosphatases and of general signalling modules. The presented computational approach is generic and may enable elucidating the effects of studied interactions also of other cellular systems under more specific conditions.

This is a *PLOS Computational Biology* Methods article.

## Introduction

Accurately reconstructing signal transduction pathways is central to elucidate cellular mechanisms. For a true model of a living system, one would ultimately need to represent thousands of individual reactions each requiring several parameters to describe them. Efforts such as Reactome (www.reactome.org) [1] attempt to capture the correct relationships in a System Biology ready format, though it will clearly be decades before sufficient data are available to model pathways systematically using (for example) ordinary differential equations. There are currently few efforts aimed at completing the parameter space for such modelling exercises. Such efforts are limited by a lack of high-throughput techniques for biochemical experiments, as these would require isolation of molecules on a scale that is currently unimaginable. There are, however, many high-throughput experiments to study gene function, protein-protein interactions and the phenotypic consequences of interfering with genes or proteins within biological systems. Information about protein-protein interactions (PPIs) may serve as the basis to assemble signal transduction pathways on a large scale [2], [3]. With the help of manual curators, experimentally validated information about direct PPIs and functional relations have been extracted from literature and collected in well-established databases [4]–[7]. In addition, RNA interference (RNAi) technology has been established to study the function of single human genes and the proteins they encode. This technology was scaled up to genome-wide screens [8]. Notably, RNAi technology is very general and allows analyzing a large variety of different treatments and cell lines making it a desirable approach for large-scale inference of protein function [9], [10] [11] [12]. Low throughput experiments studying individual molecular perturbations (e.g. RNAi, gene over-expression, or chemical modulators) have allowed molecular biologists gradually (often over decades) to build up pictures of biological processes commonly termed pathways. Within pathways, interactions between molecules are typically represented as a series of activation or inhibition events, which have typically been inferred over years on the basis of painstaking over-expression or deletion studies of individual molecules. While over-simplistic, these pathways serve as an extremely useful, tried-and-tested framework to display the sum of information known and to test hypotheses about new molecules or relationships.

In this work we have attempted, in part, to deduce pathway relationships in a similar fashion though by using high-throughput datasets. Specifically, we studied phenotypes observed in HeLa cell cultures in which single genes were knocked down covering a large portion of the genome. The aim of our study was to elucidate if two interacting proteins either positively propagated a signal (activating signal) or if their interaction led to an inhibition of the signal. Hereto, we developed a new approach based on the idea that activating signals should lead to very similar RNAi knockdown phenotypes of the respective interacting proteins, whereas inhibiting signals should lead to dissimilar phenotypes. Just recently, a study on *Drosophila* came out which follows a similar concept [13]. Comparing our approach to this method showed that our method suits distinctively better for the data we analyzed (see below, Results). We used a large range of phenotype descriptors. These descriptors included features from a novel concept that employs a performance criterion of a machine learning method to estimate the similarity of pairs of individually knocked down genes. We applied this approach to cellular images of HeLa cells at standard cultivation conditions which were collected in the Mitocheck genome-wide RNAi knockdown screen [10].

## Results

### Assembling known activating, inhibiting and undefined interactions

Three non-overlapping sets of interactions were defined. The first set consisted of 5,864 known interactions that were described to be activating. They were taken from literature based data repositories and used as a reference or gold standard for activating PPIs (Act-PPIs). The second set comprised 1,006 interactions that have been reported to be inhibiting (Inh-PPIs). The third set consisted of 9,652 high-confidence PPIs supported by multiple types of evidence (see Methods) and for which no knowledge on activation or inhibition was available (Undef-PPIs, undefined PPIs). We used the latter dataset to characterize their effects (activation/inhibition). It was not part of this study to infer novel PPIs but rather the *effect* of a known interaction.

### General concept and workflow

**Figure 1:**
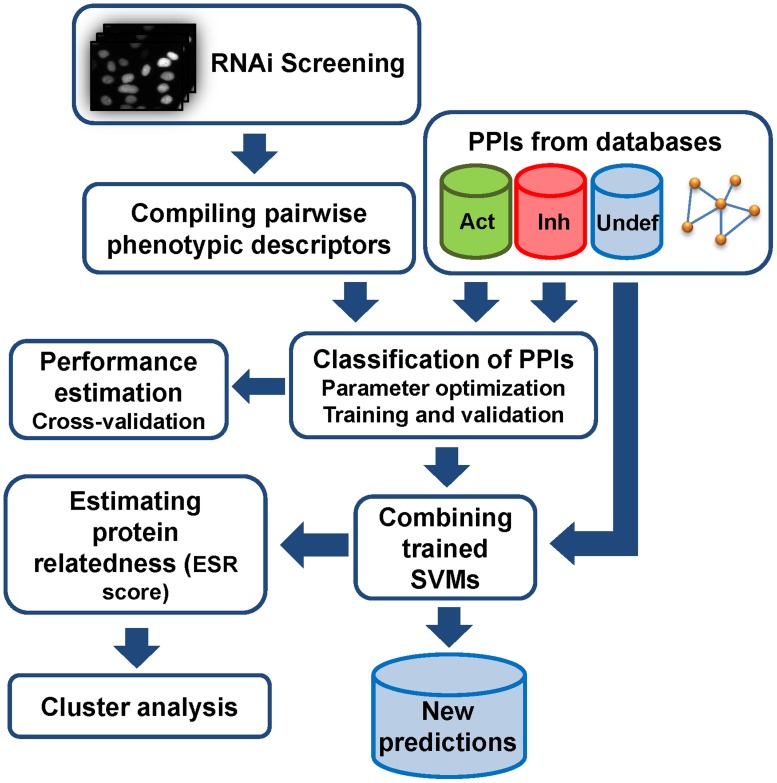
Workflow. Images of a genome-wide cellular RNAi knockdown screen (the screening data was derived from the Mitocheck project, www.mitocheck.org) were segmented and their features extracted to compile pairwise phenotype descriptors for a large set of gene pairs. These descriptors were used to train a machine learning system to discriminate activating and inhibiting PPIs taken from a reference. The performance was evaluated using cross-validation. The trained SVM models were used to predict the effects of uncharacterized PPIs. In addition, the SVM models were used to estimate similarity of the effects of proteins for all combinations of protein pairs in the network. Subsequently, this Effect Similarity Rate (ESR) was exemplarily used for clustering of functionally related protein sub-networks.

An overview of the entire workflow of our methodology is given in Figure 1. Our aim was to infer an activating effect between two protein partners of a PPI (Act-PPI) if knockdown of the corresponding genes results in a similar phenotype and to infer an inhibitory effect (Inh-PPI) if the resulting knockdown phenotypes are dissimilar. To distinguish similar from dissimilar phenotypes, we calculated a large set of different features for each of these phenotype pairs (Supplementary Table S1 lists all features):

- One feature was derived from our novel concept employing Linear Discriminant Analyses (LDAs). For each gene pair, the task of the classifier (LDA) was to distinguish images of cells with a knockdown of these genes. Good performance resulted in high accuracy indicating that the phenotypes of the two knockdowns were dissimilar (pointing to an inhibiting interaction). In contrast, weak performance indicated similar phenotypes (pointing to an activating interaction). The performance of the LDAs served as a similarity criterion and was used as the first feature (*LDA-performance-feature*).
- As further features, we employed cell counts in different states of interphase, mitosis and apoptosis and the overall cellular proliferation rates. We used the time-points and heights of the maximal counts in each class for each knocked down gene from the Mitocheck study [10] and calculated their differences between each gene of a gene-pair to derive the features for the gene pairs.
- Finally, we calculated features from phenotype similarities (employing LDA-performance from (i) and maximal counts from (ii)) to the same set of gene pairs.

All features were used to train a second set of classifiers (Support Vector Machines, SVMs) to classify gene-pairs of the set of known Act-PPIs and Inh-PPIs. Their performance was assessed employing a cross-validation procedure. The trained classifiers were subsequently used to predict the effect of undefined interactions. These predictions on the effect of the interactions were uploaded to the database HIPPIE [14]. Furthermore, the trained classifiers were used to define a similarity score (Effect Similarity Rate, ESR) indicating the prediction-concordance to other proteins in the network. ESR was high for a pair of proteins if their effects on other proteins were similar or low otherwise. As a case study, ESR was used for a clustering analysis of chemokine signaling.

### Characterizing phenotypic similarity and dissimilarity of cells using LDAs

**Figure 2:**
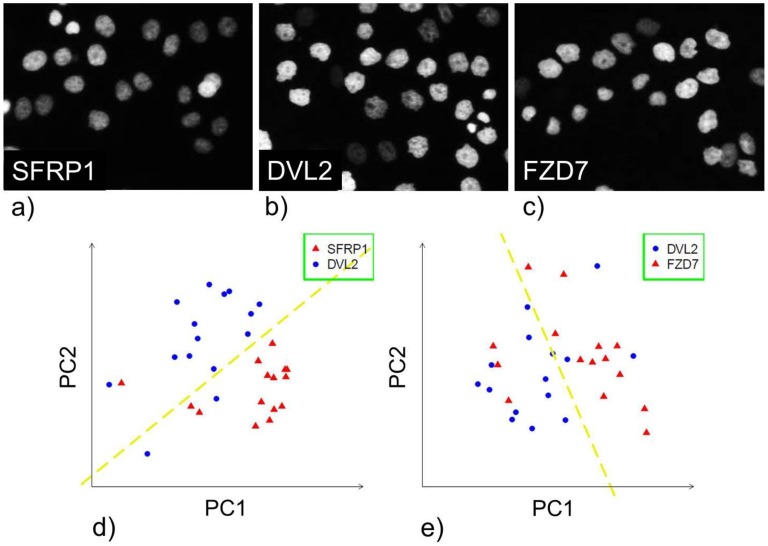
Characterization of phenotypic similarity by linear discrimination. (**a-c**) Images of cells in which *sfrp1, dvl2* or *fzd7* were knocked down, respectively. (**d**) First two principal components (PC 1 and PC 2) of the features for cells with knockdown of *sfrp1* and *dvl2*. (**e**) First two principal components of the features for cells with knockdown of *dvl2* and *fzd7*. Dotted lines sketch a linear separation.

PPIs were investigated by analyzing cell images of knockdowns of their corresponding genes. For each gene of the investigated PPIs, we took images from live HeLa cells with GFP-tagged histones for chromatin staining after knockdown of the gene. The use of LDAs for describing phenotypic similarity is exemplarily described for three sample knockdowns illustrated in Figure 2. Two of the genes, frizzled family receptor 7 (*FZD7*) and dishevelled 2 (*DVL2*), are functionally tightly related. *DVL2* is activated by *FZD7* in the Wnt signaling cascade [15]. Thus, cellular images after individual knockdown of those two genes should show a phenotypic similarity. In contrast, *SFRP1* (secreted frizzled-related protein 1) forms an inhibitory complex with the frizzled receptor and down-regulates Wnt signaling [16]. Hence, *SFRP1* and *DVL2* (or *FZD7*) should show dissimilar cellular phenotypes after knockdown. Indeed, cells after knockdown of *FZD7* and *DVL2* displayed considerably irregular nuclei membranes (Figures 2b and c). In contrast, cells after knockdown of *SFRP1* did not show these irregular patterns (Figure 2a) and were therefore better distinguishable from cells after *FZD7* and *DVL2* knockdown.

We segmented the cells of all images and calculated a broad range of texture, morphological and shape features for each cell. Feature vectors were compared for cells with knockdown of *SFRP1* and *DVL2* (dissimilar images), and for cells with knockdown of *DVL2* and *FZD7* (similar images). Figures 2d and 2e show the results for the first two principal components (containing the highest variance of the data in the feature space). Cells with knockdown of *SFRP1* were better separable from cells with knockdown of *DVL2* than cells with knockdown of *FZD7* (LDA accuracy of 89.8% and 70.6%, respectively). This shows that activating effects (*FZD7* activates *DVL2*) can lead to similar images whereas inhibiting effects (*DVL2*, *SFRP1*) can lead to dissimilar effects and this can be captured by LDA performance. Figure S1 in the supplement shows all three datasets in one principal component plot.

### Learning to distinguish activating from inhibiting PPIs

**Figure 3:**
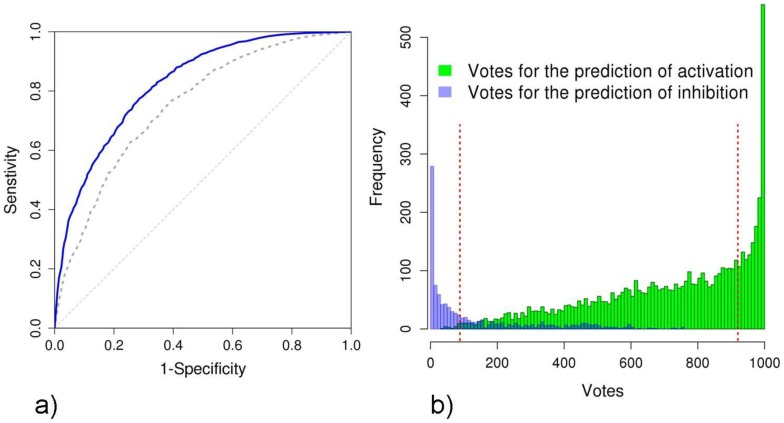
a) Receiver Operating Characteristics curves for the predictions of activation. Cross-validation results for all pathways combined (AUC  = 0.75, dashed line) and when training and validation was done for each set of pathways separately (AUC  = 0.82, solid line). **b**) Histogram of the votes for activating PPIs (green) and inhibiting PPIs (blue) when training and validation was done for each set of major signaling pathways separately. The thresholds for 80% confidence were set at 920 and 88 votes for activation and inhibition, respectively (dashed lines).

We trained 1000 Support Vector Machines to distinguish the set of Act-PPIs from the set of Inh-PPIs. Training and validation was done by cross-validation. To obtain different levels of stringency, a voting scheme was applied: a positive vote was contributed for each classifier that predicted an activating interaction. Votes from all trained SVM-classifiers were summed up to yield the predicted interaction effect and the number of votes was used to define stringency. Applying this method to all genes of all investigated signaling pathways, we yielded a receiver operating characteristic (ROC-curve) with an area under the curve (AUC) of 0.75 with respect to the activating interactions (dashed line in Figure 3a). The same performance was achieved with respect to the predictions of inhibiting interactions (Supplementary Figure S2). We considerably improved the performance using classifiers that were separately trained and validated on each of the major sets of pathways and yielded an AUC of 0.82 (solid line in Figure 3a and Supplementary Figure S2).

Figure 3b illustrates the distribution of the votes for the training data. We were particularly interested in classifiers with high stringency. At highest stringency, we yielded remarkably good precision for the interactions predicted unequivocally by all classifiers (precision: 92.9%; accuracy: 55.4%, sensitivity: 11.6%, specificity: 99.1%). Using a minimum of 90% of the votes for stringency yielded a high precision (87.8%) with considerably high specificity (94.8%), and sensitivity of 37.8%. A list of results for all investigated cutoffs is given in Supplementary Table S2.

### Comparing our method with a study published recently

We compared our approach to the method of Vinayagam and coworkers published recently [13]. They constructed a protein interaction network for *Drosophila melanogaster* using siRNA image data of other studies and predicted 6,125 effects to identify positive and negative regulators of signaling pathways and protein complexes connecting 3,352 genes. We used our sets of features (Phenotype fraction, Maxima, and Proximity features, LDA performance features), normalized the feature values and applied their correlation method on qualitative features as reported by Vinayagam *et al*. Performing a stringent filtering procedure (see Methods), we found a precision of 0.78 and a recall of 0.51 for 93 interacting pairs.

### Predicting the effect of undefined PPIs

We applied all trained SVMs (of all cross-validations) on the features of the interactions not defined as activating or inhibiting (Undef-PPIs) to predict their previously unknown effects. We obtained 508 predictions for activation and 125 predictions for inhibition with high confidence (≥90%, see Methods for details). With good confidence (≥80%), we obtained 1,548 predictions for activation and and 406 for inhibition. The results for all predictions are given in the Supplementary Table S3 and are provided in the database HIPPIE (see below).

### Functional validation using domain annotations of the interacting proteins domains

To test the ability of our approach to accurately identify true activation or inhibition events, we sought for combinations of domains in pairs of interacting proteins that were, in a textbook fashion, likely to indicate activation or inhibition events. To do this we first classified proteins according to the Pfam (pfam.sanger.ac.uk) [17] domains they contained as effectors, receptors, kinases, phosphatases and of general signalling modules (Supplementary Table S4 lists the investigated Pfam domains and their according classes) permitting proteins to belong to more than one class and also considering domains independently. For each pair of classes we then computed the fraction of predicted activating or inhibiting interactions that contained the pair, computed the ratio and a Chi-Square P-value to assess the statistical significance of the difference.

Reassuringly, significant pairs enriched in activators relative to inhibitors correspond to several text-book interactions between domains that are generally considered to be activating in nature. The pairs most enriched in activators are all indicative of activation relationships (e.g. kinase-receptor P = 9.0e-05, kinase-kinase, P = 7.7e-05) in contrast to those enriched in inhibitors which predominantly involve phosphatases, and which in pathway terms are most often inhibitory (Table 1). Applying the same test to the training set finds similar preferences (Supplementary Table S5), though also additional relationships that are not significant in the predictions (p>0.1). Notably the textbook effector-receptor, receptor-kinase and kinase-kinase relationships are also favoured among the positives and interactions with phosphatases among the negatives. In addition, we investigated statistical enrichment using protein annotation from Gene Ontology (www.geneontology.org). In line, we found considerably higher significance in enrichment of kinases for predictions of activating interactions (Act-PPIs: kinase activity: P = 3.0E-08; Inh-PPIs: kinase activity: P = 7.8E-04). We also tested for enrichment of phosphatase activities and found significance *only* for the predictions of inhibiting activations (Inh-PPIs: phosphatase activity: P = 1.9E-04).

**Table 1:** Pairs of Pfam domain sets showing significant* enrichment of predicted interactions.

Protein A class	Protein B Class	Number of activating interactions (of 1549)	Number of inhibiting interactions (of 407)	Enrichment	P-value
effector	effector	70	2	9.2	0.00012
effector	kinase	44	2	5.8	0.0054
kinase	kinase	158	16	2.6	7.7e-05
effector	receptor	74	8	2.4	0.012
kinase	receptor	178	20	2.3	9.0e-05
effector	signalling	56	8	1.8	0.096
receptor	receptor	134	20	1.8	0.013
signalling	signalling	432	142	0.8	0.0058
kinase	phosphatase	36	26	0.4	3.1e-05
phosphatase	signalling	42	36	0.3	1.8e-08
phosphatase	receptor	12	22	0.1	2.0e-10

^*^p≤0.1 only; t≥1 for both pos/neg - no zeroes.

### Interactions with receptors give the best predictions

Because the interactions represent a broad functional survey, we investigated the performance of our predictions for specific subsets of the signaling pathways. Hereto, we assigned three major (non-overlapping) sets comprising 1) receptors which are initiating the signaling processes in the cell, 2) further, central (highly connected) proteins in the pathways, and 3) transcription factors as the signals' destinations. For each of these subsets, we selected interactions containing at least one node of these sets. We yielded highest performance for the receptors (AUC  = 0.89). Performance was average for the set of highly connected proteins (AUC  = 0.78), and lowest for the transcription factors (AUC  = 0.59), which may reflect their promiscuous functions.

### A clustering analysis reveals a functional similarity of a subset of chemokine receptors

The complex network of interactions contains functional information at multiple levels of resolution. We investigated this network through clustering of similar effects to reveal functional similarity of coherent subgroups. For this, the Effect Similarity Rate (ESR) was defined estimating the concordance of the votes of the genes in a particular interaction to other connected proteins in the network. A high ESR score indicated a high number of other genes that show the same activation/inhibition predictions to both genes of the pair (for details see Methods).

More specifically, we investigated chemokine receptors and their signaling interactions. Chemokine receptors are cytokine receptors and they initiate signaling to regulate the response of a cell in diverse cellular processes including inflammation and immune surveillance. In cancer cells, this response is often crucially disturbed leading to aberrant signaling and finally induced proliferation [18]. Therefore chemokine receptors have been widely studied as potential drug targets. Chemokine receptor signaling has mainly been described for leukocytes, but it is also observed in epithelial cells [19] (we investigated HeLa cells which originate from an epithelial tumour).

**Figure 4:**
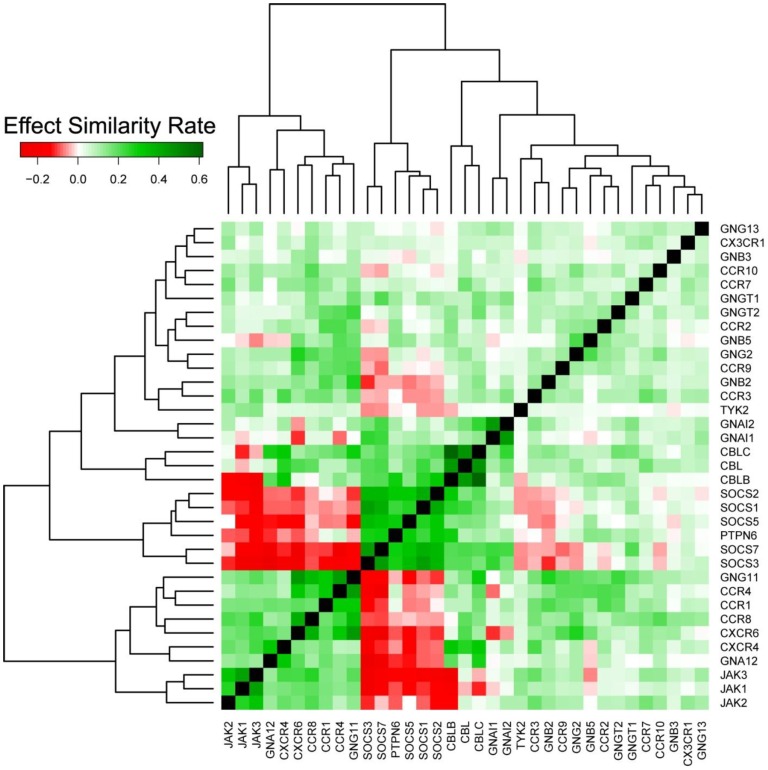
Clustering of the chemokine receptors and their interactors.

To elucidate the functional interplay between chemokine receptors and their direct downstream interactors, we selected a set of genes coding for the chemokine receptors themselves, JAK1, JAK2, JAK3 (JAKs) and TYK2 as the receptors' direct downstream signaling targets activating the JAK/STAT signaling cascade [20], G-proteins mediating PI3-kinase/AKT signaling of chemokines (chemokine receptors are G-protein coupled receptors[21]), and the SOCS family inhibiting cytokine signaling [22]. Indeed, genes with functional similarity clustered into subgroups based on their ESR scores (Figure 4). Such a clustering was not evident when not using our predictions (see Supplementary Figure S3). As expected, protein pairs between SOCS and their target groups had very low ESR reflecting their inhibiting effects. In particular, the set of SOCS showed very high dissimilarity to JAKs and TYK2 confirming their inhibitory role for these signaling cascades. In contrast, genes among the set of SOCS themselves showed very high similarity and clustered tightly together.

A subset of CCRs consisting of CCR1, CCR4, CCR8, CXCR4 and CXCR6 (denoted as CCR-subset in the following) showed high similarity among itself, high similarity to their downstream interactors of JAKs and high dissimilarity to their inhibitors of the SOCS subset. Based on the clustering in Figure 4, we divided the set of CCRs into two subsets: the CCR-subset and the rest of investigated CCRs (CCR2, CCR3, CCR7, CCR9, CCR10, CX3CR1). The ESR scores of pairs within the CCR-subset were significantly higher than the ESRs of pairs within the other CCRs (P  = 3.56E-04). Moreover, the ESR scores of the pairs within the CCR-subset were significantly higher than ESR scores of the pairs between the CCR-subset and the other CCRs (P = 7.93E-04). We further validated that that these five CCR genes form a subgroup by co-expression analysis. We used a large set of 5,896 gene expression profiles from microarrays (76 different studies from the CAMDA competition, www.ebi.ac.uk/arrayexpress, accession E-TABM-185) and compared the expression correlation of pairs within the CCR-subset with the correlation of pairs within the set of the other CCRs. We found a significantly higher correlation of expression in the CCR-subset compared with the other investigated CCRs (P = 6.08E-05) evidencing higher functional relatedness of the CCRs in the subset. A functional interpretation of the association between the CCR1, CCR4, CXCR4 and CXCR6 proteins included in the identified subset is given in Supplementary Text S1.

### Availability of the data and integration into a database

**Figure 5:**
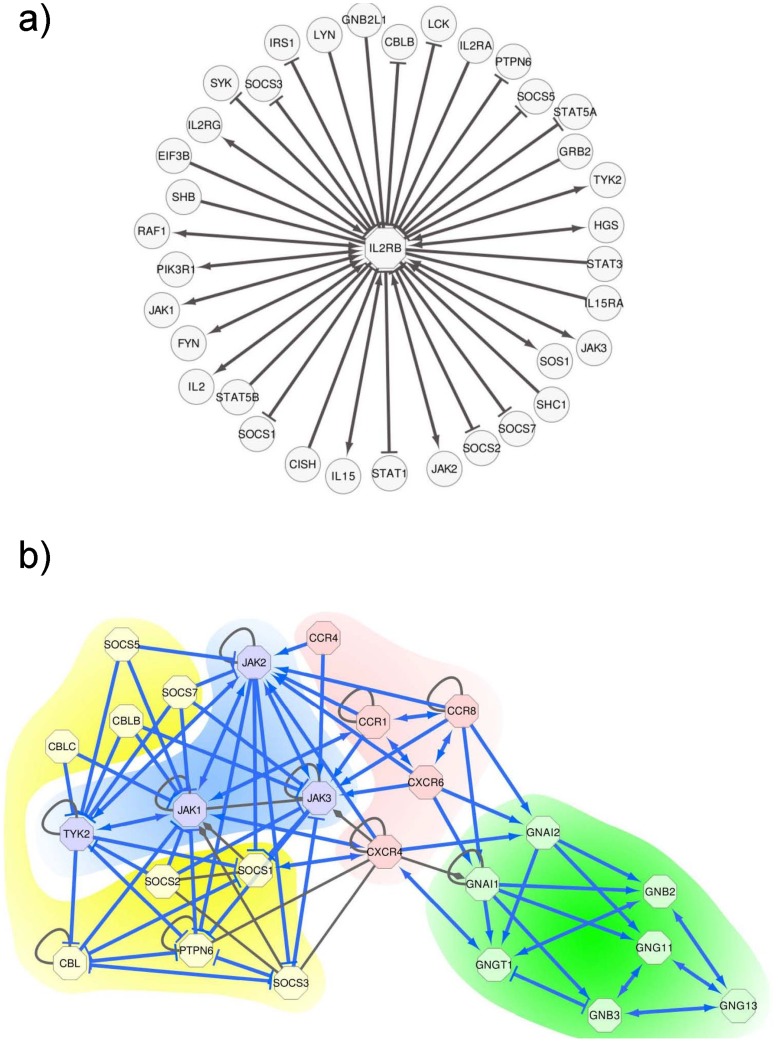
Querying and visualizing inhibitory and activating interactions with HIPPIE. **a**) Querying HIPPIE with the IL2RB, a receptor that transduces IL2 signals in immune response, reveals 35 interaction partners from which 24 are associated with an effect prediction (HIPPIE indicates activations by arrows and inhibitions by bars). 11 of these effects are also found in KEGG and are correctly reproduced by our approach. 2 interactions for which we predict an effect are listed in KEGG but have no effect assigned there (IL2, SOS1). 11 predicted effects are not annotated at all as interactions in KEGG (FYN, HGS, IL15, IL2RG, IRS1, LCK, PIK3R1, RAF1, STAT1, STAT5A, SYK). Many of these interaction partners are, however, organized in cytokine-related pathways and, thus, demonstrate the potential of our approach to not just reproduce KEGG annotations but also to detect novel and meaningful interaction effects. **b**) We uploaded interactions for which we could predict an activating or inhibiting effect within the CCR-subset and its direct interactors to HIPPIE (blue edges). Using the default HIPPIE output-options, HIPPIE extended the query set of interactions with additional interactions between the input proteins. The newly added interactions are colored in grey. We also enabled the option from the HIPPIE menu to display interaction directions as defined by KEGG. Arrows are unidirectional if the directions were known (from KEGG) and bidirectional otherwise. HIPPIE uses diamond-shaped (grey) arrows to indicate that an interaction has associated a direction but no effect. We manually highlighted CCRs in pink, JAKs in blue, SOCS in yellow and G-proteins in green. The depicted predictions for activation and inhibition are correct (except for the interactions between GNGT1 and GNB3, and between SOCS1 and CXCR4): SOCS are inhibiting JAKS, CCRs, G-proteins, all other protein pairs are activating. It is to note that in our representation, edges with two arrows (bidirectional edges) indicate that the direction of the effect is not known. They *do not* indicate a bi-direction of an interaction of e.g. a simple feedback loop (in which A activates B which in turn activates A).

We made our predictions of the interaction effects available via the web based database HIPPIE [14]. HIPPIE collects human PPIs from the major public PPI databases and associates them with confidence scores reflecting their experimental reliability. Several analysis and visualization features allow to generate functional- and expression-specific networks and to highlight pathway information within these networks. In addition to the initial effect predictions on the high confidence set of PPIs, we extended our predictions to all PPIs from HIPPIE and added the effects of previously undefined interactions to the HIPPIE database. In total we annotated 10,960 of the 115,189 interactions currently stored in HIPPIE (version 1.5) with our effect predictions. These data can be accessed in different ways. When the graphical output mode of HIPPIE is chosen, the effect prediction is visually encoded by edges terminating in either arrows (activation) or bars (inhibition) (making use of Cytoscape web [23]). Alternatively, the generated effect-associated networks can be exported into different formats. Figure 5 gives two examples (Figure 5b without background coloring) illustrating how to explore the set of predicted PPIs with HIPPIE. HIPPIE can be used at http://cbdm.mdc-berlin.de/tools/hippie (specifically, to query the effect of a single gene pair: selecting “network query”, typing the two gene symbols into the empty box, selecting “show in browser – text”, “Layers: 0” and “show predicted effect” at “Inhibitory or activating effect”).

### Software implementation and availability

The method is implemented in a software package for R (www.r-project.org, version 2.15.0 or higher) which runs on a Linux machine. It is freely available at http://www.ichip.de/software/InteractionAnalyzer.html.

## Discussion

Accurately reconstructing signal transduction cascades is fundamental for understanding the regulatory mechanisms of a cell. In contrast to former large-scale investigations aiming at discovery of new signal transduction interactions, our goal was to improve the characterization of the effect of such interactions. We investigated changes in cellular phenotypes of HeLa cells after gene knockdown. We observed images of all possible pairs of genes (images of single gene knockdowns) encoding the corresponding protein partners of a PPI and predicted activating and inhibiting effects of such a PPI by similarity and dissimilarity of the cells after gene knockdown. For this, we set up a similarity criterion derived from a novel concept that employs a performance feature of a classifier (LDA) to distinguish cells of similar and dissimilar phenotypes. Similar or dissimilar phenotypes of knocked down gene pairs resulted in low or high performance of this classifier, respectively. This performance feature was employed together with an elaborated set of further features by a second set of classifiers (SVMs) distinguishing activating from inhibiting effects. Cross-validation showed that our approach was well suited for this task. Applying all trained classifiers to yet uncharacterized protein interactions, we inferred 1,954 new predictions with good confidence (≥80%) of activating (1,548) and inhibiting (406) effects in the signaling cascades. Investigating domain-domain interactions of these predictions showed that the textbook effector-receptor, receptor-kinase and kinase-kinase relationships were favoured among the interactions predicted as activating and interactions with phosphatases among the interactions predicted as inhibiting. These results provide strong support that the method proposed here is effective in identifying true activation and inhibition events. No information about protein function or domain composition was incorporated in the predictions in any way, but nevertheless, the approach has apparently reproduced general textbook relationships between proteins or domains effectively. It is highly encouraging that such relationships can be rediscovered using information only about the cellular phenotype and we expect that this suggests a great potential use for this technique as more large RNAi databases become available. For example, when using our approach for a clustering analysis of chemokine receptors, we identified high similarity for a subset of five chemokines including CCR4 and CXCR4 (CCR1, CCR4, CCR8, CXCR4 and CXCR6). Interestingly, in HeLa cells, it was shown that CXCR4 was cross-desensitized by a ligand for CCR4. In chemotaxis, CKLF1 is an activator of CCR4, and SDF1 is an activator of CXCR4. CKLF1 inhibits the effect of SDF1, which is mediated by CCR4, as SDF1 can be rescued, acting as an activator of chemotaxis after blocking CCR4 [24]. Together with our findings of similar knockdown phenotypes of these receptors, we suggest that both receptors may signal through very similar downstream cascades. It would be intriguing to follow up experimentally on this by e.g. testing such cross-desensitization also for CCR1, CCR8 and CXCR6 employing their specific ligands and following their signaling cascades. Further, one can expect interesting differences when compared for different cell types, e.g. originating from monocytes, T-cells, stern cells and epithelial cells (as HeLa cells).

Using the ESR score yielded clustering of well defined gene groups like JAKs/TYK, CCR, SOCS, G-proteins, and the functional interplay of chemokine receptors to their direct downstream interactors. The idea of ESR is that we did not compare two sets directly but followed a guilt-by-association concept before comparing the gene pairs. Knockdown phenotypes just being dissimilar may not necessarily have resulted from an inhibitory effect, but may also have resulted from loss of rather distinct functions of the two proteins, in particular if the proteins are involved in a complex inter-connected network with several other proteins participating in multiple functions. This may explain the false positives we got. In addition, an activating or inhibiting role of a protein may be context-specific and also rewiring of protein interaction networks can be observed under different conditions and in different cell types. To address this, the here described approach can be readily applied to images obtained on different cell lines and pathological states (e.g. cancer types), which might shape out more fine grained and distinctive effects of disease and tissue specific protein interactions. It is to note that we inferred activation and inhibition effects leading to causal relationships, such as protein "A" activates "B" or "A" inhibits "B". The proposed approach did not infer directionality of the interactions. Such directionality inference may be done using e.g. the approach of Vinayagam *et al*. [25]. We used images of siRNA induced knockdown genes. To reduce off-target effects we used data consisting of siRNA knockdowns with two different siRNA constructs. Still, off-target effects cannot entirely be ruled out. We compared our method with the method by Vinayagam and coworkers published recently [13]. Our method was superior on the data we analyzed, in respect to the prediction performance (us: 82% AUC, e.g. 79% precision comes along with 61% recall, in comparison to Vinayagam *et al*.: 78% precision, 51% recall), but also to the exploitation of the data (us: 100%, them: 5%). To note, Vinayagam and coworkers' method worked very well on the datasets they analyzed. We plan to elaborate on the data of the Drosophila phenotypes in a future project.

Our method is generic, in particular with respect to characterizing the effect of protein interactions, and annotated the activating and inhibiting effect of protein interactions for a large set of to date non-characterized interactions. The approach may enable to yield cell type specific effects when applied to the specific cellular system under study. The software and new predictions are available online at http://www.ichip.de/software/InteractionAnalyzer.html and http://cbdm.mdc-berlin.de/tools/hippie, respectively and are likely to reveal interesting insights across a wide variety of datasets.

## Materials and Methods

### Defining the gold standard, the PPI repository for new predictions and the sets of signaling pathways

Three (non-overlapping) sets of interactions were defined: 1) Activation PPIs (Act-PPIs) consisted of well-known interactions that have been described as activating. They were taken from the literature-based data repositories of the Kyoto Encyclopedia of Genes and Genomes (KEGG [4]), Biocarta (from Biocarta Inc., PPIs were retrieved from the R-package Graphite [26]) and MetaCore (from GeneGo Inc.). 2) Inhibitory PPIs (Inh-PPIs) comprised PPIs for which an inhibiting interaction has been reported in the same databases. 3) Undefined PPIs (Undef-PPIs) consisted of interactions for which there was no information in these databases on activation or inhibition. These interactions were taken from HIPPIE [14] and from the computationally inferred data repository of the Search Tool for the Retrieval of Interacting Genes and Proteins (STRING, version 9.0 [27]). We selected putative PPIs with a STRING score ≥800. To select only physically interacting protein pairs with high reliability, we restricted the selection to protein pairs with domains predicted to interact. For this, the corresponding genes were mapped to their proteins for which their Pfam entries were collected using the Sequence Retrieval System of HUSAR [28], [29]. A gene pair was added to our list of undefined PPIs if (in addition to STRING score ≥800) the corresponding proteins had at least one interacting domain as listed in the database DOMINE [30]. We trained and validated our system with PPIs of ten major sets of signaling pathways in KEGG: Signal Transduction, Transport and Catabolism, Cell Motility, Cell Growth and Death, Cell Communication, Immune System, Endocrine System, Circulatory System, Nervous System and Development. The gene lists for selecting the undefined PPIs were also taken from these pathways.

### Segmenting the cell nuclei and calculating features for the interaction classifiers

The analyzed knockdown images were obtained from the Mitocheck Database (www.mitocheck.org). Generation of these images is explained in detail elsewhere [10]. Briefly, morphological changes in the nuclei of HeLa cell clones, which were stably transfected with the GFP-tagged histone 2B, were tracked by fluorescence imaging after transient transfection of siRNAs in a high-throughput screen. The cells were distributed on cell microarrays (one well live cell imaging chambers with coverslip bottoms) printed with transfection-ready siRNAs, and the chromosome/nuclear morphology was visualized in real-time. One image typically contains more than 100 nuclei with an average diameter of approximately 30 pixels in G1 phase. All images have a grey value depth of 16 bit and a spatial resolution of 1344×1024 pixels. Each image sequence consists of 96 time points over 48 hours. We performed segmentation and feature extraction by using an automated image processing system as described earlier [9]. Each single cell nucleus was segmented by Otsu thresholding and characterized by morphological descriptors, e.g. Haralick texture features, Zernike moments, granularity features, object-and edge-related features, grey-scale invariants, number of cells and pixels calculated. Haralick texture features were computationally very intensive and hence computed using Graphical Processing Units (GPUs, see Supplementary Text S2). All features were used to distinguish different phenotypes of cells. Each single cell was classified into four morphological classes, consisting of interphase, apoptosis, mitosis, and shape (cluster of cells) by using Support Vector Machines (SVMs). The classifier was trained to distinguish these four phenotype classes with a training set of 621 manually annotated nuclei (details are given in the supplement, Text S2).

Counts of each of these classes for a gene knockdown served to calculate the first set of features (fraction features) for classifying the protein interactions. The fractions of each phenotype were computed with respect to the number of cells in an image for each knocked down gene. As well, overall cell counts, median and standard deviation of grey level intensities of the cells, were calculated. To obtain features for a *pair* of knocked down genes, we calculated the absolute value of the differences of the features between the genes of the respective pair.

A further set of features (maxima features) was derived from the study by Neumann and co-workers [10]. We extracted the phenotype scores of seven morphological phenotypes from the Mitocheck database (www.mitocheck.org). They comprised the features mitotic delay, binuclear, polylobed, grape, large, dynamic change, and cell death. Their scores were derived from the time point with the maximum difference of cell counts between the negative controls and the cells of the respective class (of one of the seven morphological phenotypes). In addition, also the time points for these maxima were taken as features. To obtain features for a pair of knocked down genes, we calculated the absolute value of the differences between the features of each gene of the respective pair.

The LDA-performance feature was calculated by using the classification method of linear discriminant analysis (LDA). This analysis tried to separate cells in which the two respective genes were knocked down. The LDA was trained on 60% of the images for each cell knockdown, and tested on the remaining 40%. To obtain expressed phenotype information and reduce computational time, only the last 40 time points from the entire time series of images were used. Testing resulted in true positive (tp), true negative (tn), false positive (fp) and false negative (fn) predictions. From these, the accuracy ((tp+tn)/(tp+tn+fp+fn)) was derived, which served as a further similarity criterion for the interaction classifiers. As we calculated similarity features for all gene pairs it was necessary to further reduce the computational complexity. We reduced the texture features by selecting the most discriminative features. LDAs were applied to 89,925 randomly selected gene pairs, the features ranked according to their regression coefficients, the ranks summed up for each feature and the 50 features (see Table S9 in Text S2) with the highest ranks selected as representing features with the largest regression coefficients (the threshold was selected after initial trials).

We calculated another set of features (we denoted these as proximity features) basing on the LDA-performance feature and the maxima features. For the proximity-features, we compared the similarity of each of the knocked down genes of the gene pair to a set of reference genes. The selection of the reference genes is described in the supplement (Supplementary Text S3). The rational of this was that similar phenotypes of the knocked down gene pair should lead to similar phenotypes of each of the knocked down gene with respect to the reference genes. This similarity was estimated using the LDA-performance-feature and the Euclidean distance of the set of maxima features.

### Training and validation of the interaction classifiers

Based on the pairwise phenotype descriptors, we trained a set of 1,000 classifiers with the interactions of the gold standard of activating (Act-PPI) and inhibiting PPIs (Inh-PPI). This was done for all genes of all investigated pathways. In addition, we performed this training and validation also for each observed major set of signaling pathways to improve the prediction performance (by selecting interactions of genes for which both genes belonged to the respective major set of signaling pathway of KEGG). To assess the performance of the classifiers, a 10-times-10-fold cross-validation was performed and this was repeated ten times. In each cross-validation, activating and inhibiting PPIs were randomly split into ten equally sized, non-overlapping subsets. Nine subsets were concatenated and used to train the classifiers. Testing was done with the remaining subset (test set). The performance was measured on the test set by comparing the predictions with the true class labels. In our dataset, the sizes of the two classes (Act-PPI and Inh-PPI) differed considerably. Therefore, data stratification was performed. In each training subset, ten SVM classifiers were trained with an equal number (between 80 and 400, depending on the number of training samples of the class with the lower number of samples of each investigated pathway-set) of randomly selected activating and inhibiting PPIs. To optimize the parameters for the SVM-classifiers, an inner five fold cross-validation was embedded for each training cycle. We used SVMs with a radial basis kernel and optimized cost of false classification (*C*) and kernel width (γ) employing a grid search with *C* = 2^n^ and γ = 2^n^ for n є {−5, −4, … 4, 5}. Each pair of C and γ was tested and the pair with the lowest validation error (the average number of misclassified samples) was chosen and used for training an SVM on the complete training dataset. LIBSVM [31] was used for all SVM calculations.

To obtain performance estimates for different stringency settings, the classifiers were combined into an ensemble and a voting scheme was applied. 100 repetitions of the cross-validation procedure yielded 100 predictions for each PPI of the test sets and these were used for defining stringency. Each SVM contributed one vote. The highest stringency was derived for predictions for which all classifiers voted for either activation or inhibition. Several cutoffs on the number of votes were calculated yielding a receiver operator characteristic (ROC) curve and its area under the curve (AUC) was used as a performance criteria. This was done for predictions of activation and inhibition separately (yielding two ROC curves). To predict effects for uncharacterized interactions, all 1,000 trained classifiers were employed as an ensemble classifier and the same voting scheme was applied. For the list of new predictions, we selected PPIs with a confidence level ≥0.8 (predicted precision ≥0.8 using the stratification as for the training data, see Supplementary Text S4). For new predictions for which both genes belonged to one of the major sets of signaling pathways, we took the confidence level of the classifiers, which were trained and validated on the major sets of pathways separately (yielding cutoffs of 920 and 88 votes for activation and inhibition, respectively), see Supplementary Tables S2a and S2b, votes were averaged for PPIs which were part of more than one set of major pathways). For all other predictions we used the classifiers trained and validated on the whole gene set (≥995 and ≤25 votes, see supplementary Tables S2c and S2d).

### Defining gene groups coding for transcription factors, receptors and highly connected proteins

To analyze the performance on different subsets of the signaling network, we selected genes from three different groups: receptors, central proteins (highly connected) and transcription factors. Receptors were selected if a gene contained the GO term "receptor activity". After manual refinement, we selected 256 genes coding for receptors. 38 central proteins were selected due to their highest betweenness centrality and node degree higher than 20 of a network constructed by the PPIs of HIPPIE [14] (version 1.5) of our investigated gene-set. 58 human genes of the network coding for transcription factors were assembled from Transfac and Jasper as described in our recent study [32].

### Calculating the Effect Similarity Rate

To define a similarity criterion for a clustering analysis, we compared the effect of each of the knocked-down genes of a gene-pair (gene i, gene j) to all other investigated knocked-down genes (gene k). An effect was denoted similar if, for a gene pair *i,j*, the votes of both gene *i* to gene *k* and gene *j* to gene *k* were in concordance. More specifically, the threshold for a similar effect was set to 80% confidence and therefore defined as ≥920 votes for activation and ≤88 votes for inhibition from the classifiers that were separately trained and validated on the major sets of pathways; and as ≥995 votes for activation and ≤25 votes for inhibition from the SVMs that were trained and validated on the whole gene sets. *Vice versa*, if the votes from gene *i* to gene k predicted activation and from gene *j* to gene k inhibition, the effect was denoted dissimilar. This comparison was done for all genes *k*, *k*∈{all genes of the pathway set \ {*i*,*j*}, and the ratio of similar and dissimilar effects was used to compute the Effect Similarity Rate (ESR, high ESR  =  high number of other genes that show the same activation/inhibition predictions to both genes of the pair). ESR was computed from the difference of the percentage of similar and dissimilar effects X by ESR = tanh(5X) bringing ESR into a descriptive range between −1 and 1 (−1: gene *i* and *j* are dissimilar with respect to all other genes; +1: gene *i* and *j* are similar with respect to all other genes).

### Gene expression analysis

The gene expression data comprised datasets from the CAMDA 2007 competition (ArrayExpress, www.ebi.ac.uk/arrayexpress, accession E-TABM-185) and was analyzed as described recently [33]. Briefly, it contained expression values for 13,069 genes with 4,064 primary human tissue samples of 76 experimental conditions collected from a wide range of human cancer types with normal and disease tissue samples. The data was normalized using the RMA method as implemented in the *affy* R-package (www-r-project.org). Pearson's correlation coefficients were computed for each pair and condition. Finally, we used the average of correlation coefficients of all 76 datasets as the correlation coefficient for each pair of genes and this served to show the similarity of these genes.

### Comparing our method with a study published recently

We compared our approach to the approach of Vinayagam and coworkers [13]. We used our sets of features (Phenotype fraction, Maxima, and Proximity features; LDA performance feature: see below) and employed a z-transformation to obtain significance cuttoffs. As in Vinayagam *et al*., the cutoffs of −1.5 and 1.5 were used. If z-scores were equal or larger than 1.5, the genes got the feature “positive regulator”, if z-scores were equal or less than −1.5, the genes got the feature “negative regulator”. Positive and negative regulators were annotated with the values +1 and −1, respectively. Using these calculated features, a phenotype matrix was constructed, with the genes in the rows and the phenotypes in the columns. For an interacting protein, we classified for each feature either a positive correlation (both +1 or both −1) or a negative correlation (one feature value was +1 and the other one was −1). As the LDA performance features were calculated for pairs of genes and not single genes, they were directly transformed into correlation features. LDA performance features were transformed to z-scores and the same cutoffs used for negative and positive regulators as for the other features. The number of positive and negative correlations of each pair was counted. The sign score of Vinayagam *et al*. was used to calculate the score of each interacting pair. We used the same criterion as Vinayagam *et al*. and selected only interacting pairs with two or more matching features (positive and negative correlation) for validation.

## Supporting Information

Figure S1 Characterization of phenotypic similarity by linear discrimination. (a-c) Images of cells in which *srfp1, dvl2* or *fzd7* were knocked down, respectively. (d) First two principal components (PC 1 and PC 2) of the features for cells with knockdown of *sfrp1, fzd7* and *dvl2*. Dotted lines sketch a linear separation. Separation of knockdown of dvl2 and fzd7 was more difficult for the classifier (24 true predictions, 6 false predictions from the example images shown here), compared to separating sfrp1 and dvl2 (27 true predictions, 3 false predictions).
(EPS)

Figure S2 a) Receiver Operating Characteristics for the predictions of inhibition. Cross-validation results for machines that were trained and validated on all pathways combined (AUC  = 0.75, dashed line) and for machines that were trained and validated on each set of pathways separately (AUC  = 0.82, solid line).
(EPS)

Figure S3 Clustering of the chemokine receptors and their interactors without taking our predictions into account. The clustering was performed as described in Methods of the main text but using randomized predictions. To get this clustering, we randomized the predictions and re-computed the ESR score again using only the correct information of the annotation from the training data.
(EPS)

Table S1 Phenotype features.
(DOC)

Table S2 Validation results of the predictions of activation and inhibition for different thresholds.
(DOC)

Table S3 Predictions for activation and inhibition.
(XLS)

Table S4 Functional classes of the investigated domains from Pfam.
(DOC)

Table S5 Pairs of Pfam domain sets showing significant enrichment of interactions from the training set.
(DOC)

Text S1 Functional interpretation of the identified chemokine subset.
(DOC)

Text S2 Segmentation, feature extraction and classification of cell images.
(DOC)

Text S3 Identifying reference genes for the proximity features.
(DOC)

Text S4 Defining a confidence score for the new predictions.
(DOC)
