# Supplementary material for: Characterizing Protein Interactions Employing a Genome-Wide siRNA Cellular Phenotyping Screen
Source: PLoS Comput Biol. 2014 Sep 25;10(9):e1003814. doi: 10.1371/journal.pcbi.1003814 (PMC4178005; doi:10.1371/journal.pcbi.1003814)
Supplement: Table S1 — Phenotype features. (DOC) [file pcbi.1003814.s004.doc]

# Table S1 Phenotype features

| **Phenotype fraction features** |
| --- |
| Distance computed from the fraction of Apoptotic cells |
| Distance computed from the fraction of Interphase cells |
| Distance computed from the fraction of Mitosis cells |
| Distance computed from the fraction of Cluster cells |
| Distance computed from the median of mean of cell intensities |
| Distance computed from the median of standard deviation of cell intensities |
| Distance computed from the median of number of cell images pixels |
| Distance computed from the median of number of cells |
| Distance computed from the cell proliferation rates |
| **LDA performance feature** |
| **Maxima features** |
| Distance computed from the maximum scores of the mitotic delay phenotype |
| Distance computed from the maximum scores of the binuclear phenotype |
| Distance computed from the maximum scores of the polylobed phenotype |
| Distance computed from the maximum scores of the grape phenotype |
| Distance computed from the maximum scores of the large phenotype |
| Distance computed from the maximum scores of the dynamic change phenotype |
| Distance computed from the maximum scores of the cell death phenotype |
| Distance computed from the time points of the maxima of the mitotic melay phenotype |
| Distance computed from the time points of the maxima of the binuclear phenotype |
| Distance computed from the time points of the maxima of the polylobed phenotype |
| Distance computed from the time points of the maxima of the grape phenotype |
| Distance computed from the time points of the maxima of the large phenotype |
| Distance computed from the time points of the maxima of the dynamic change phenotype |
| Distance computed from the time points of the maxima of the cell death phenotype |
| **Proximity features** |
| Distance computed from reference gene (1-5) using the phenotype profiles |
| Distance computed from reference gene (1-5) using the LDA performance feature |

# 
