# Supplementary material for: Characterizing Protein Interactions Employing a Genome-Wide siRNA Cellular Phenotyping Screen
Source: PLoS Comput Biol. 2014 Sep 25;10(9):e1003814. doi: 10.1371/journal.pcbi.1003814 (PMC4178005; doi:10.1371/journal.pcbi.1003814)
Supplement: Table S2 — Validation results of the predictions of activation and inhibition for different thresholds. (DOC) [file pcbi.1003814.s005.doc]

# Table S2 Validation results of the predictions of activation and inhibition for different thresholds

# Activating predictions for training and validation on each set of pathways separately

| **Votes** | **Accuracy** | **Precision** | **Sensitivity** | **Specificity** |
| --- | --- | --- | --- | --- |
| 100 | 0.55 | 0.93 | 0.12 | 0.99 |
| 95 | 0.63 | 0.90 | 0.29 | 0.97 |
| 90 | 0.66 | 0.88 | 0.38 | 0.95 |
| 85 | 0.68 | 0.84 | 0.45 | 0.92 |
| 80 | 0.70 | 0.82 | 0.51 | 0.89 |
| 75 | 0.71 | 0.81 | 0.56 | 0.87 |
| 70 | 0.72 | 0.79 | 0.61 | 0.84 |
| 65 | 0.73 | 0.77 | 0.65 | 0.80 |
| 60 | 0.73 | 0.75 | 0.69 | 0.77 |
| 55 | 0.74 | 0.75 | 0.73 | 0.75 |
| 50 | 0.74 | 0.73 | 0.77 | 0.72 |
| 45 | 0.74 | 0.72 | 0.80 | 0.69 |
| 40 | 0.74 | 0.71 | 0.82 | 0.66 |
| 35 | 0.73 | 0.69 | 0.85 | 0.62 |
| 30 | 0.73 | 0.68 | 0.87 | 0.59 |
| 25 | 0.72 | 0.67 | 0.90 | 0.55 |
| 20 | 0.72 | 0.65 | 0.92 | 0.51 |
| 15 | 0.70 | 0.63 | 0.94 | 0.45 |
| 10 | 0.67 | 0.61 | 0.97 | 0.38 |
| 5 | 0.63 | 0.58 | 0.98 | 0.29 |
| 1 | 0.57 | 0.54 | 1.00 | 0.15 |

1. **Inhibiting predictions for training and validation on each set of pathways separately**

| **Votes** | **Accuracy** | **Precision** | **Sensitivity** | **Specificity** |
| --- | --- | --- | --- | --- |
| 0 | 0.57 | 0.97 | 0.15 | 1.00 |
| 5 | 0.64 | 0.93 | 0.31 | 0.98 |
| 10 | 0.68 | 0.91 | 0.39 | 0.96 |
| 15 | 0.70 | 0.88 | 0.46 | 0.94 |
| 20 | 0.72 | 0.86 | 0.52 | 0.92 |
| 25 | 0.73 | 0.84 | 0.56 | 0.90 |
| 30 | 0.73 | 0.82 | 0.60 | 0.87 |
| 35 | 0.74 | 0.80 | 0.63 | 0.84 |
| 40 | 0.74 | 0.79 | 0.66 | 0.82 |
| 45 | 0.74 | 0.77 | 0.69 | 0.79 |
| 50 | 0.74 | 0.75 | 0.72 | 0.76 |
| 55 | 0.74 | 0.73 | 0.76 | 0.72 |
| 60 | 0.73 | 0.71 | 0.78 | 0.69 |
| 65 | 0.73 | 0.70 | 0.81 | 0.65 |
| 70 | 0.72 | 0.68 | 0.84 | 0.60 |
| 75 | 0.71 | 0.66 | 0.87 | 0.55 |
| 80 | 0.70 | 0.64 | 0.90 | 0.50 |
| 85 | 0.68 | 0.62 | 0.92 | 0.44 |
| 90 | 0.66 | 0.60 | 0.95 | 0.36 |
| 95 | 0.62 | 0.57 | 0.97 | 0.27 |
| 100 | 0.50 | 0.50 | 1.00 | 0.00 |

1. **Activating predictions for all pathways combined**

| **Votes** | **Accuracy** | **Precision** | **Sensitivity** | **Specificity** |
| --- | --- | --- | --- | --- |
| 100 | 0.54 | 0.86 | 0.09 | 0.99 |
| 95 | 0.60 | 0.79 | 0.26 | 0.93 |
| 90 | 0.62 | 0.77 | 0.35 | 0.89 |
| 85 | 0.64 | 0.75 | 0.42 | 0.86 |
| 80 | 0.66 | 0.75 | 0.47 | 0.85 |
| 75 | 0.67 | 0.74 | 0.51 | 0.82 |
| 70 | 0.67 | 0.73 | 0.55 | 0.79 |
| 65 | 0.68 | 0.71 | 0.59 | 0.76 |
| 60 | 0.68 | 0.70 | 0.63 | 0.73 |
| 55 | 0.68 | 0.68 | 0.67 | 0.69 |
| 50 | 0.68 | 0.68 | 0.70 | 0.67 |
| 45 | 0.69 | 0.67 | 0.74 | 0.64 |
| 40 | 0.69 | 0.66 | 0.76 | 0.61 |
| 35 | 0.68 | 0.65 | 0.79 | 0.57 |
| 30 | 0.67 | 0.63 | 0.82 | 0.53 |
| 25 | 0.67 | 0.62 | 0.85 | 0.49 |
| 20 | 0.66 | 0.61 | 0.88 | 0.45 |
| 15 | 0.65 | 0.60 | 0.90 | 0.39 |
| 10 | 0.63 | 0.58 | 0.93 | 0.33 |
| 5 | 0.60 | 0.56 | 0.96 | 0.25 |
| 0 | 0.50 | 0.50 | 1.00 | 0.00 |

1. **Inhibiting predictions for all pathways** combined

| **Votes** | **Accuracy** | **Precision** | **Sensitivity** | **Specificity** |
| --- | --- | --- | --- | --- |
| 0 | 0.56 | 0.91 | 0.13 | 0.99 |
| 5 | 0.61 | 0.85 | 0.27 | 0.95 |
| 10 | 0.63 | 0.82 | 0.34 | 0.93 |
| 15 | 0.65 | 0.80 | 0.41 | 0.90 |
| 20 | 0.67 | 0.78 | 0.46 | 0.87 |
| 25 | 0.67 | 0.76 | 0.49 | 0.84 |
| 30 | 0.67 | 0.74 | 0.53 | 0.81 |
| 35 | 0.68 | 0.73 | 0.58 | 0.78 |
| 40 | 0.69 | 0.72 | 0.62 | 0.76 |
| 45 | 0.68 | 0.70 | 0.64 | 0.73 |
| 50 | 0.69 | 0.69 | 0.67 | 0.70 |
| 55 | 0.68 | 0.67 | 0.70 | 0.66 |
| 60 | 0.69 | 0.67 | 0.74 | 0.63 |
| 65 | 0.68 | 0.65 | 0.77 | 0.59 |
| 70 | 0.67 | 0.64 | 0.79 | 0.55 |
| 75 | 0.66 | 0.62 | 0.83 | 0.50 |
| 80 | 0.65 | 0.61 | 0.85 | 0.46 |
| 85 | 0.64 | 0.59 | 0.87 | 0.41 |
| 90 | 0.62 | 0.57 | 0.90 | 0.34 |
| 95 | 0.59 | 0.55 | 0.94 | 0.24 |
| 100 | 0.50 | 0.50 | 1.00 | 0.00 |
