# Supplementary material for: Characterizing Protein Interactions Employing a Genome-Wide siRNA Cellular Phenotyping Screen
Source: PLoS Comput Biol. 2014 Sep 25;10(9):e1003814. doi: 10.1371/journal.pcbi.1003814 (PMC4178005; doi:10.1371/journal.pcbi.1003814)
Supplement: Table S4 — Functional classes of the investigated domains from Pfam. (DOC) [file pcbi.1003814.s007.doc]

# Table S4. Functional classes of the investigated domains from Pfam

| Functional classes | Pfam domains |
| --- | --- |
| Effectors | cEGF, CSF-1, CXCR4_N, EGF_2, EGF_CA, EGF, EPO_TPO, FGF, GM_CSF, IL12, IL13, IL15, IL1, IL2, IL3, IL4, IL5, IL6, IL7, IL8, Insulin, Laminin_EGF, Laminin_G_2, LIF_OSM, TGF_beta, TGF_beta_GS |
| Receptors | 7tm_1, 7tm_2, 7tm_3, Activin_recp, C1_1, C1-set, C2, C2-set_2, C2-set, CD20, CD45, CD4-extracel, Ephrin_lbd, EpoR_lig-bind, fn1, fn2, fn3, ig, IL4Ra_N, IL6Ra-bind, Interfer-bind, I-set, Kringle, Lep_receptor_Ig, Tcell_CD4_Cterm, TCR_zetazeta, TNFR_c6, V-set |
| Kinases | PI3_PI4_kinase, PI3_PI4_kinase, Pkinase_C, Pkinase_C, Pkinase, Pkinase, Pkinase_Tyr, Pkinase_Tyr |
| Phosphatases | DSPc, Exo_endo_phos, Metallophos, Y_phosphatase |
| General signalling modules | 14-3-3, Arrestin_C, Arrestin_N, BTK, Cyclin_C, Cyclin_N, IRS, Metallophos, PDZ, PH, PI3Ka, PI3K_C2, PI3K_rbd, PI3_PI4_kinase, PI-PLC-X, PI-PLC-Y, Pkinase_C, Pkinase, Pkinase_Tyr, PP2C_C, PP2C, RA, RGS, Rho_Binding, SH2, SH3_1, SH3_2, SOCS_box, WW, Y_phosphatase |
