# Supplementary material for: Characterizing Protein Interactions Employing a Genome-Wide siRNA Cellular Phenotyping Screen
Source: PLoS Comput Biol. 2014 Sep 25;10(9):e1003814. doi: 10.1371/journal.pcbi.1003814 (PMC4178005; doi:10.1371/journal.pcbi.1003814)
Supplement: Table S5 — Pairs of Pfam domain sets showing significant enrichment of interactions from the training set. (DOC) [file pcbi.1003814.s008.doc]

# Table S5. Pairs of Pfam domain sets showing significant^*^ enrichment of interactions from the training set

| Protein A class | Protein B class | Number of activating interactions  (of 5857) | Number of inhibiting interactions (of 1013) | Enrichment | P-value |
| --- | --- | --- | --- | --- | --- |
| effector | kinase | 164 | 0 | 57 | 7.0e-08 |
| effector | receptor | 654 | 4 | 28 | 5.5e-27 |
| receptor | receptor | 410 | 4 | 18 | 3.4e-16 |
| kinase | receptor | 918 | 24 | 6.6 | 6.1e-30 |
| effector | signalling | 218 | 8 | 4.7 | 1.3e-06 |
| kinase | kinase | 604 | 68 | 1.5 | 0.00037 |
| signalling | signalling | 1906 | 260 | 1.3 | 1.4e-05 |
| kinase | signalling | 1544 | 226 | 1.2 | 0.0065 |
| phosphatase | receptor | 126 | 64 | 0.34 | 8.2e-14 |
| phosphatase | signalling | 246 | 236 | 0.18 | 0 |
| kinase | phosphatase | 110 | 216 | 0.088 | 0 |

^*^p≤0.1 only; t≥1 for both pos/neg - no zeroes
