# Supplementary material for: Characterizing Protein Interactions Employing a Genome-Wide siRNA Cellular Phenotyping Screen
Source: PLoS Comput Biol. 2014 Sep 25;10(9):e1003814. doi: 10.1371/journal.pcbi.1003814 (PMC4178005; doi:10.1371/journal.pcbi.1003814)
Supplement: Text S1 — Functional interpretation of the identified chemokine subset. (DOC) [file pcbi.1003814.s009.doc]

# Supplementary Text S1: Functional interpretation of the identified chemokine subset

Cytokine receptors act as dimers or even higher order oligomers . In recent years, several studies have found evidence for common action of the gene products from the subset of CCRs put forward in this study. Seidl and coworkers investigated gene expression profiles of chemokines using real-time PCR for melanocytes, melanoma cell lines and primary and metastatic melanoma. They found that the pair of CXCR4 and CCR1 is consistently expressed in these melanoma cells, and that CXCR6 was expressed *de novo* in primary melanomas and melanoma metastases . The receptors CXCR4 and CXCR6 were reported in several studies to play a predominant role in the development and progression of solid tumors. CXCR4 and CXCR6 interact with tumor cells by activating the AKT/mTor signaling pathway . Further, CXCR4 is known to activate cancer progression by the JAK/STAT pathway and CXCR4 is associated with poor prognosis in cervical cancer patients . CXCR4 is highly expressed in gynecological tumors and CXCR6 in inflammation associated tumors, and both play an important role in growth, proliferation, invasion, and metastasis of epithelial ovarian carcinoma . CXCR6 was found to be highly involved in metastasis and progression of several types of cancer . Prostate cancer uses the CXCL16/CXCR6 axis for development and cancer aggressiveness . Our results demonstrate the common phenotypes of CXCR4 and CXCR6. This is in accordance to a study by Hu and coworkers who showed that CXCR6 and CXCR4 were expressed in similar proportions in malignant prostate tumors and benign prostate hyperplasia tissue, and both of them were highly expressed in the malignant tissue . CCR1, CCR4 and CXCR4 were reported to function on human platelets activated in patients infected with the human immunodeficiency virus (HIV) and may be commonly involved in inflammatory or allergic responses . Interestingly, in HeLa cells, it was shown that CXCR4 was cross-desensitized by a ligand for CCR4. For chemotaxis, CKLF1 is an activator of CCR4, and SDF1 an activator of CXCR4. CKLF1 could inhibit the effect of SDF1, and this was mediated by CCR4 as SDF1 could be rescued acting as an activator of chemotaxis after blocking CCR4 . Together with our findings that these receptors have similar knockdown phenotypes, we hypothesize that both receptors may signal through very similar downstream cascades. This may be potentiated when the other receptor is absent leading to the same phenotypic shape regardless of the expressed receptor. In summary, these evidences suggest similar functions for CCR1, CCR4 CXCR4 and CXCR6 of our subgroup of chemokine receptors.

# References

1. G. Milligan, G protein-coupled receptor dimerisation: molecular basis and relevance to function. *Biochim Biophys Acta* **1768**, 825 (2007); published online EpubApr (

2. M. A. Lemmon, J. Schlessinger, Cell signaling by receptor tyrosine kinases. *Cell* **141**, 1117 (2010); published online EpubJun (

3. H. Seidl, E. Richtig, H. Tilz, M. Stefan, U. Schmidbauer, M. Asslaber, K. Zatloukal, M. Herlyn, H. Schaider, Profiles of chemokine receptors in melanocytic lesions: de novo expression of CXCR6 in melanoma. *Human pathology* **38**, 768 (2007); published online EpubMay (10.1016/j.humpath.2006.11.013).

4. L. Deng, N. Chen, Y. Li, H. Zheng, Q. Lei, CXCR6/CXCL16 functions as a regulator in metastasis and progression of cancer. *Biochim Biophys Acta* **1806**, 42 (2010); published online EpubAug (10.1016/j.bbcan.2010.01.004).

5. A. J. Vila-Coro, J. M. Rodríguez-Frade, A. M. D. Ana, M. C. Moreno-Ortíz, C. Martínez-A, M. Mellado, The chemokine SDF-1alpha triggers CXCR4 receptor dimerization and activates the JAK/STAT pathway. *FASEB J* **13**, 1699 (1999); published online EpubOct (

6. J. Kodama, Hasengaowa, T. Kusumoto, N. Seki, T. Matsuo, Y. Ojima, K. Nakamura, A. Hongo, Y. Hiramatsu, Association of CXCR4 and CCR7 chemokine receptor expression and lymph node metastasis in human cervical cancer. *Ann Oncol* **18**, 70 (2007); published online EpubJan (

7. L. Guo, Z.-M. Cui, J. Zhang, Y. Huang, Chemokine axes CXCL12/CXCR4 and CXCL16/CXCR6 correlate with lymph node metastasis in epithelial ovarian carcinoma. *Chin J Cancer* **30**, 336 (2011); published online EpubMay (

8. L. Deng, N. Chen, Y. Li, H. Zheng, Q. Lei, CXCR6/CXCL16 functions as a regulator in metastasis and progression of cancer. *Biochim Biophys Acta* **1806**, 42 (2010); published online EpubAug (

9. H. K. Ha, W. Lee, H. J. Park, S. D. Lee, J. Z. Lee, M. K. Chung, Clinical significance of CXCL16/CXCR6Â expression in patients with prostate cancer. *Mol Med Report* **4**, 419 (2011).

10. W. Hu, X. Zhen, B. Xiong, B. Wang, W. Zhang, W. Zhou, CXCR6 is expressed in human prostate cancer in vivo and is involved in the in vitro invasion of PC3 and LNCap cells. *Cancer science* **99**, 1362 (2008); published online EpubJul (10.1111/j.1349-7006.2008.00833.x).

11. K. J. Clemetson, J. M. Clemetson, A. E. Proudfoot, C. A. Power, M. Baggiolini, T. N. Wells, Functional expression of CCR1, CCR3, CCR4, and CXCR4 chemokine receptors on human platelets. *Blood* **96**, 4046 (2000); published online EpubDec 15 (

12. Y. Zhang, L. Tian, Y. Zheng, H. Qi, C. Guo, Q. Sun, E. Xu, D. Ma, Y. Wang, C-terminal peptides of chemokine-like factor 1 signal through chemokine receptor CCR4 to cross-desensitize the CXCR4. *Biochemical and biophysical research communications* **409**, 356 (2011); published online EpubJun 3 (10.1016/j.bbrc.2011.05.047).
