# Supplementary material for: Characterizing Protein Interactions Employing a Genome-Wide siRNA Cellular Phenotyping Screen
Source: PLoS Comput Biol. 2014 Sep 25;10(9):e1003814. doi: 10.1371/journal.pcbi.1003814 (PMC4178005; doi:10.1371/journal.pcbi.1003814)
Supplement: Text S2 — Segmentation, feature extraction and classification of cell images. (DOC) [file pcbi.1003814.s010.doc]

**Supplementary Text S2: Segmentation, feature extraction and classification of cell images**

**Segmentation and feature extraction**

To analyze images of the siRNA screen, an automated system was employed which we described previously (1). Briefly, a quadratic sliding window was used to calculate local thresholds for different image regions. The local threshold was only calculated if the variance within the window reached a pre-defined threshold (2000), otherwise a global threshold was used. The window consisted of an outer region of 15 pixels in which the thresholds were computed and an inner region of 2 pixels in which the thresholds were applied. The window was shifted by the length of its inner region. Global and local thresholds were calculated using Otsu thresholding. After segmentation, quantitative image features were extracted from the image for each single cell. They comprised granularity features, object- and edge-related features, tree-structured wavelet features, Haralick texture features, grey-scale invariants and Zernike moments. In total, we computed 361 features for each cell nucleus. With these features, single-cell images were classified into the classes interphase, mitosis, apoptosis and cell clusters. Only images of cells classified as interphase were used in further analyses. It would have taken several weeks or months to compute Haralick texture features from all image data, therefore, we employed general-purpose graphics processing units (GPUs) to speed up the computation of co-occurrence matrices and Haralick texture features. A massive parallel software version for GPUs was designed and implemented for this purpose. Computation time was shortened by a factor of 32 on a single node of a cluster in comparison to a pre-existing optimized CPU software version (2).

**Description of the image features**

We extracted a set of image features of each single cell. Table S6 shows the number of extracted features for each feature set. All these features were described in details in Harder et al, 2008 (1) and are briefly described in the following. The features based on size and shape and included the area (A, number of pixels of the segmented object), the contour length p of a segmented object, the circularity c (c=p2/A) and Feret’s diameter (calliper length) which is the maximum distance between any two contour pixels. Additional edge-related features were computed by applying Laplace and Sobel filters to the image and subsequent thresholding. The features based on geometric moments including, e.g., the distance of the gravity center to the bounding box center, and the ratio of second order central moments μ20 and μ02. Wavelet-based features are based on a recursive subdivision of the image into different frequency channels (3). These features were computed for four subdivision cycles. Zernike moments use complex Zernike polynomials as a moment basis set. We computed Zernike moments up to degree 12 and used the moment’s magnitudes as features, which are invariant to rotation as proposed, e.g., in (4), resulting in 49 features. Granularity features considered the difference of gray values of pixel pairs in a certain distance under a certain angle. As feature values the mean and standard deviation of the maximum differences were computed over the whole image. Here, distances of one to ten pixels considering eight directions were used. Gray scale invariants (5) combine sets of neighboring pixels using local kernel functions of different scales, which is followed by integration over the whole image. We used two different kernels with radii of 2, 4, 16, and 32 pixels. Haralick texture features (6) are based on co-occurrence matrices of pixel pairs with a certain distance under a certain angle. We used distances of one to five pixels and four different angles resulting in 20 co-occurrences matrices. For each of such matrices 13 features were computed, including, e.g., contrast, entropy, and angular second moment.

# Table S6 Sets of features

| **Feature set** | **Total number** |
| --- | --- |
| Haralick texture features | 260 |
| Zernike moments | 49 |
| Granularity features | 21 |
| Object-related features | 8 |
| Edge-related features | 3 |
| Gray scale invariants | 10 |
| Tree-structured wavelets | 2 |

To classify cell nuclei into phenotype classes, a representative set of single cell nuclei was taken from different images of randomly selected knockdowns and time points and this was manually classified by experts. Four classes were assigned: 1) interphase, 2) mitosis, 3) cell death, and 4) shape (clustered nuclei). The total number of manually classified cell objects was 775 (see Table S7).

# Table S7 Training and test sets for classifying the cellular phenotypes

| **Classes** | **Training set** | **Test set** | **Total** |
| --- | --- | --- | --- |
| **Interphase** | 252 | 62 | 314 |
| **Mitosis** | 172 | 43 | 215 |
| **Apoptosis** | 89 | 22 | 111 |
| **Shape** | 108 | 27 | 135 |
| **Total** | 621 | 154 | 775 |

We split the available samples for each class randomly into training and test sets at a ratio of 4:1. We trained an SVM classifier with a Gaussian radial basis function (RBF) kernel (*C*=1 and $\gamma$=0.00358) on the training data set. The samples of the test set were classified into the four classes. This was done by a cross-validation: we repeated the classification step applying ten times random sampling on the whole data set. Performance of the classification from training and test sets is shown in Table S8a and S8b, respectively. This yielded an overall accuracy for the training set of 99.6% and the test set of 96.6%. Misclassifications occurred mostly between the classes mitosis and apoptosis which were even for experts difficult to distinguish.

#

# Table S8 a) Classification results of the images of the training sets

| **True class** | **Classifier output** | | | | **Accuracy^*^** |
| --- | --- | --- | --- | --- | --- |
|  | **Interphase** | **Mitosis** | **Apoptosis** | **Shape** |  |
| Interphase | **252** | 0 | 0 | 0 | **100.00%** |
| Mitosis | 0 | **172** | 0 | 0 | **100.00%** |
| Apoptosis | 0 | 2.1 | **86.9** | 0 | **97.6%** |
| Shape | 0.2 | 0 | 0 | **107.8** | **99.8%** |

^*^The overall accuracy is 99.6% (618.7/621).

# b) Classification results of the images of the test sets

| **True class** | **Classifier output** | | | | **Accuracy^*^** |
| --- | --- | --- | --- | --- | --- |
|  | **Interphase** | **Mitosis** | **Apoptosis** | **Shape** |  |
| Interphase | **61.1** | 0 | 0.9 | 0 | **98.5%** |
| Mitosis | 0 | **41.9** | 1.1 | 0 | **97.4%** |
| Apoptosis | 0 | 3 | **18.9** | 0.1 | **85.9%** |
| Shape | 0 | 0 | 0.1 | **26.9** | **99.6%** |

^*^ The overall accuracy is 96.6% (148.8/154).

# Table S9 Set of selected 50 features

| **Feature** | **Specific description** | **Orientation** | **Pixel distance** |
| --- | --- | --- | --- |
| Haralick texture feature | Angular second moment | 0° | 3 |
| Haralick texture feature | Angular second moment | 90° | 2 |
| Haralick texture feature | Angular second moment | 135° | 1 |
| Haralick texture feature | Contrast | 0° | 5 |
| Haralick texture feature | Contrast | 45° | 4 |
| Haralick texture feature | Contrast | 135° | 3 |
| Haralick texture feature | Correlation | 0° | 2 |
| Haralick texture feature | Correlation | 90° | 1 |
| Haralick texture feature | Correlation | 90° | 5 |
| Haralick texture feature | Variance | 0° | 4 |
| Haralick texture feature | Variance | 135° | 2 |
| Haralick texture feature | Inverse difference moment | 0° | 1 |
| Haralick texture feature | Inverse difference moment | 45° | 5 |
| Haralick texture feature | Inverse difference moment | 90° | 4 |
| Haralick texture feature | Sum difference average | 0° | 3 |
| Haralick texture feature | Sum difference average | 45° | 2 |
| Haralick texture feature | Sum difference average | 135° | 1 |
| Haralick texture feature | Sum variance | 45° | 4 |
| Haralick texture feature | Sumentropy | 0° | 2 |
| Haralick texture feature | Sumentropy | 90° | 5 |
| Haralick texture feature | Sumentropy | 135° | 4 |
| Haralick texture feature | Entropy | 45° | 3 |
| Haralick texture feature | Entropy | 90° | 2 |
| Haralick texture feature | Difference variance | 0° | 1 |
| Haralick texture feature | Difference variance | 90° | 4 |
| Haralick texture feature | Difference entropy | 45° | 2 |
| Haralick texture feature | Difference entropy | 135° | 5 |
| Haralick texture feature | Information Measure I | 90° | 3 |
| Haralick texture feature | Information Measure I | 135° | 2 |
| Haralick texture feature | Information Measure II | 45° | 1 |
| Haralick texture feature | Information Measure II | 135° | 4 |
| Zernike moment | Degree = 4 | Angular = 0 |  |
| Zernike moment | Degree = 5 | Angular = 3 |  |
| Zernike moment | Degree = 6 | Angular = 0 |  |
| Zernike moment | Degree = 6 | Angular = 6 |  |
| Zernike moment | Degree = 8 | Angular = 4 |  |
| Zernike moment | Degree = 8 | Angular = 6 |  |
| Zernike moment | Degree = 9 | Angular = 1 |  |
| Zernike moment | Degree = 9 | Angular = 3 |  |
| Zernike moment | Degree = 9 | Angular = 7 |  |
| Zernike moment | Degree = 9 | Angular = 9 |  |
| Zernike moment | Degree = 10 | Angular = 4 |  |
| Zernike moment | Degree = 10 | Angular = 8 |  |
| Zernike moment | Degree = 10 | Angular = 10 |  |
| Zernike moment | Degree = 11 | Angular = 1 |  |
| Zernike moment | Degree = 11 | Angular = 3 |  |
| Zernike moment | Degree = 11 | Angular = 11 |  |
| Zernike moment | Degree = 12 | Angular = 0 |  |
| Zernike moment | Degree = 12 | Angular = 2 |  |
| Zernike moment | Degree = 12 | Angular = 12 |  |

**References**

1. N. Harder, R. Eils, K. Rohr, Automated classification of mitotic phenotypes of human cells using fluorescent proteins. *Methods Cell Biol* **85**, 539 (2008).

2. M. Gipp, G. Marcus, N. Harder, A. Suratanee, K. Rohr, R. König, R. Männer, Haralick's Texture Features Computed by GPUs for Biological Applications. *IAENG International Journal of Computer Science* **36**, 66 (2009); published online Epub February

3. T. Chang, C. J. Kuo, Texture analysis and classification with tree-structured wavelet transform. *IEEE Trans Image Process* **2**, 429 (1993).

4. M. V. Boland, M. K. Markey, R. F. Murphy, Automated recognition of patterns characteristic of subcellular structures in fluorescence microscopy images. *Cytometry* **33**, 366 (1998); published online EpubNov 1 (

5. H. Burkhardt, S. Siggelkow, *Invariant Features in Pattern Recognition -- Fundamentals and Applications*. (Wiley, 2001), pp. 269-307.

6. R. M. Haralick, Statistical and structural approaches to texture. Proceedings of the IEEE **67**, 5, 786 (1979).

# 
