# Supplementary material for: Characterizing Protein Interactions Employing a Genome-Wide siRNA Cellular Phenotyping Screen
Source: PLoS Comput Biol. 2014 Sep 25;10(9):e1003814. doi: 10.1371/journal.pcbi.1003814 (PMC4178005; doi:10.1371/journal.pcbi.1003814)
Supplement: Text S3 — Identifying reference genes for the proximity features. (DOC) [file pcbi.1003814.s011.doc]

# Supplementary Text S3 Identifying reference genes for the proximity features

For each feature (LDA-performance-feature and the profile of maxima-features), we selected a set of five reference genes to be mutually highly different. To reduce the complexity of the problem, we performed a pre-selection and selected thirty genes which showed the highest standard variation of the corresponding feature in respect of all investigated genes. For the selected genes, the features were used as distances for all possible pairs of genes. The problem of finding *k* genes (k=5) out of the pre-selected genes being as (mutually) distant as possible was formulated as a quadratic problem:

$\max\sum d_{ij}x_{i}x_{j} ,$ (1)

$\begin{matrix} subject to : \sum_{i} x_{i} \leq k, \\ x_{i}, x_{j} \in\left\{0,\left.1\right\}\right., \\ i= 1,\ldots N. \end{matrix}$ (2)

$d_{ij}$ is the distance of genes $x_{i}$ and $x_{j}$. This problem was transformed into the linear problem:

$\max\sum d_{ij}x_{ij} ,$ (3)

(4)

This yielded the genes *hsh2d, ptgir, kiss1r, tle4* and *stip1* for the LDA-performance-feature and *lef1, ncoa1, ears2, cit* and *kif1*a for the maxima feature profiles.

# 
