# Supplementary material for: Characterizing Protein Interactions Employing a Genome-Wide siRNA Cellular Phenotyping Screen
Source: PLoS Comput Biol. 2014 Sep 25;10(9):e1003814. doi: 10.1371/journal.pcbi.1003814 (PMC4178005; doi:10.1371/journal.pcbi.1003814)
Supplement: Text S4 — Defining a confidence score for the new predictions. (DOC) [file pcbi.1003814.s012.doc]

# Supplementary Text S4 Defining a confidence score for the new predictions

The predictions may vary between 0 and 100 votes. We computed the confidence score on the predictions as the ratio of correctly predicted interactions (TP/(TP+FN)) for each number of votes of the validation data separately . We calibrated the confidence values using a cubic polynomial regression model,

$c=\beta_{0}+\beta_{1}v+\beta_{2}v^{2}+\beta_{3}v^{3}$, (5)

In which v is the number of votes, β_i_ the fitting parameters and c the calibration function for the confidence scores. β_i_ were fitted for activation and inhibition separately (using the *lm* function from the R-package stats) yielding $\beta_{0}$=0.04256, $\beta_{1}$=0.02036, $\beta_{2}$=-3.151E-04, $\beta_{3}$=1.993E-06 for activation and $\beta_{0}$=0.9574, $\beta_{1}$=-0.02036, $\beta_{2}$=3.151E-04, $\beta_{3}$=-1.993E-06 for inhibition for the system trained and validated on the sets of pathway separately; and $\beta_{0}$=0.166, $\beta_{1}$=0.01358, $\beta_{2}$=-1.776E-04, $\beta_{3}$=1.057E-06 for activation and $\beta_{0}$=0.834, $\beta_{1}$=-0.01358, $\beta_{2}$=1.776, $\beta_{3}$=-1.057E-06 for inhibition for combined pathways.
